# Desmoplastic Small Round Cell Tumor of Stomach

**DOI:** 10.1155/2013/907136

**Published:** 2013-06-06

**Authors:** Ahmed Abu-Zaid, Ayman Azzam, Asma AlNajjar, Hussa Al-Hussaini, Tarek Amin

**Affiliations:** ^1^College of Medicine, Alfaisal University, P.O. Box 50927, Riyadh 11533, Saudi Arabia; ^2^Department of General Surgery, Faculty of Medicine, Alexandria University, Alexandria 21526, Egypt; ^3^Department of Pathology and Laboratory Medicine, King Faisal Specialist Hospital and Research Center (KFSH&RC), P.O. Box 3354, Riyadh 11211, Saudi Arabia; ^4^Department of Surgical Oncology, King Faisal Specialist Hospital and Research Center (KFSH&RC), P.O. Box 3354, Riyadh 11211, Saudi Arabia

## Abstract

Desmoplastic small round cell tumor (DSRCT) is an extremely uncommon, highly aggressive, and malignant mesenchymal neoplasm of undetermined histogenesis. Less than 200 case reports have been documented in literature so far. Herein, we report a 26-year-old otherwise healthy female patient who presented with a 1-month history of epigastric pain. On physical examination, a palpable, slightly mobile, and tender epigastric mass was detected. All laboratory tests were normal. A chest, abdominal, and pelvic contrast-enhanced computed tomography (CT) scans showed a 3.8 × 7.2 × 8.7 cm ill-defined mass, involving gastric fundus and extending into gastric cardia and lower gastroesophageal junction. It was associated with multiple enlarged gastrohepatic lymph nodes; the largest measured 1.2 cm. There was no evidence of ascites or retroperitoneal or mesenteric lymphatic metastases. Patient underwent total gastrectomy with D2 lymphadenectomy, splenectomy, and antecolic Roux-en-Y esophagojejunal anastomosis. Histopathological examination revealed coexpression of mesenchymal, epithelial, and neural markers. The characteristic chromosomal translocation (t(11; 22)(p13; q12)) was demonstrated on fluorescence in situ hybridization (FISH) technique. Diagnosis of DSRCT of stomach was confirmed. Patient received no postoperative radiotherapy or chemotherapy. A postoperative 3-month followup failed to show any recurrence. In addition, a literature review on DSRCT is included.

## 1. Introduction

Desmoplastic small round cell tumor (DSRCT) is an extremely uncommon, highly aggressive, and malignant mesenchymal neoplasm of undetermined histogenesis [1]. Less than 200 case reports have been documented in literature so far [2]. DSRCT has a distinctive tendency to affect young males in their 2nd and 3rd decades of life with a male-to-female ratio of 4 : 1 [3]. Peritoneal cavity is the most frequent site of involvement and often associated with diffuse multiple peritoneal implants [4].

Diagnosis of DSRCT is exceptionally challenging. Clinical signs and symptoms, as well as radiological findings, are nonspecific and similar to other intra-abdominal primary neoplasms [5]. Microscopically, DSRCT is made up of distinct collections of small round blue tumor cells surrounded by plentiful desmoplastic fibrous stroma [5]. Collective expression of mesenchymal, epithelial and neural markers [3, 4] and its association with a distinctive chromosomal reciprocal translocation (t(11; 22)(p13; q12)) [6] yield definitive diagnosis of DSRCT.

DSRCT has an extremely poor prognosis with an overall 5-year survival rate of nearly 15% [7]. Nemours combined aggressive treatment regimens were attempted; however, neither curative outcome nor notable impact on long-term survival was achieved [2, 8]. Further optimal management protocols have yet to be explored. 

Herein, we report a 26-year-old otherwise healthy female patient who presented with a 1-month history of epigastric pain.

## 2. Case Report

A 26-year-old otherwise healthy female patient presented to King Faisal Specialist Hospital and Research Center with a 1-month history of epigastric pain. Systemic review was remarkable for night sweating and weight loss of 7 kg. On physical examination, a palpable, slightly mobile, and tender epigastric mass was detected. All laboratory tests including complete blood count, renal, bone, hepatic, and coagulation profiles, lactate dehydrogenase (LDH), carcinoembryonic antigen (CEA), alfa-feto protein (AFP), CA 19-9, and CA 12-5 were normal.

**Figure 1:**
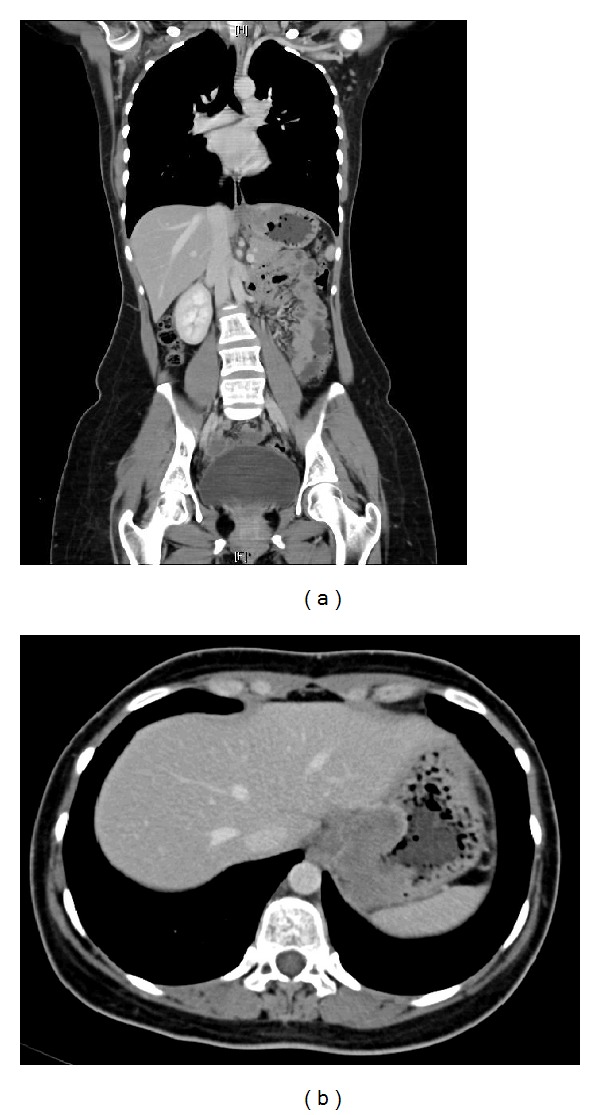
Coronal (a) and transverse (b) chest, abdominal, and pelvic contrast-enhanced computed tomography (CT) scans: showing a 3.8 × 7.2 × 8.7 cm ill-defined mass, involving gastric fundus and extending into gastric cardia and lower gastroesophageal junction. It was associated with multiple enlarged gastrohepatic lymph nodes. There was no evidence of ascites or retroperitoneal or mesenteric lymphatic metastases.

**Figure 2:**
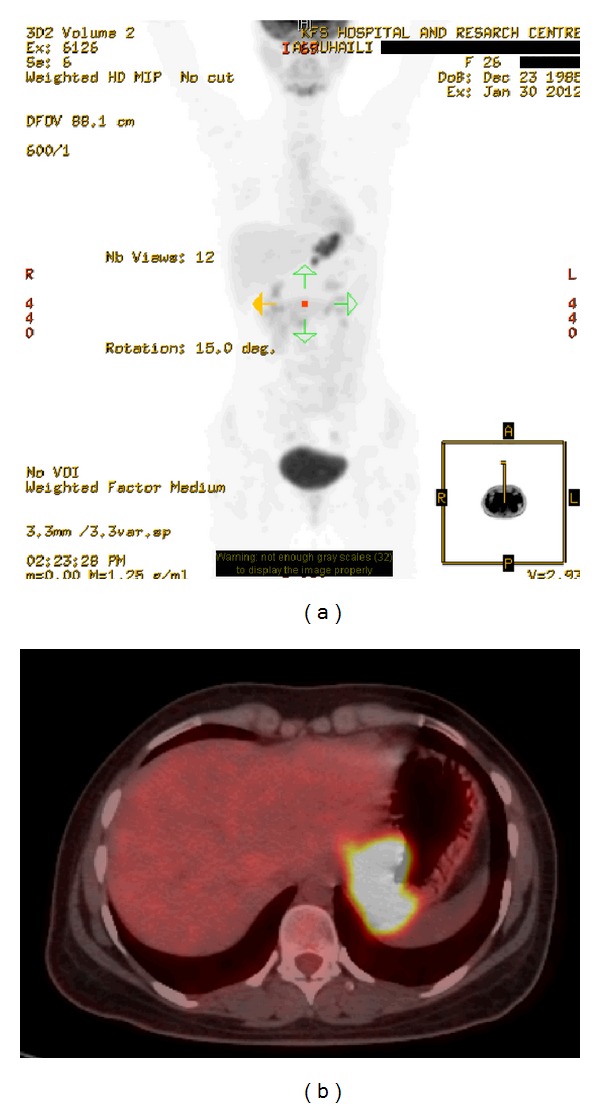
Positron emission tomography (PET) (a) and transverse-section positron emission tomography-computed tomography (PET-CT) (b) scans: showing hypermetabolic fluorodeoxyglucose- (FDG-) avid mass lesion in gastric fundus and extending into gastric cardia and lower gastroesophageal junction. In addition, it was associated with a few hypermetabolic FDG-avid lesions in the gastrohepatic junction, indicating lymph node metastases. No evidence of distant metastasis was identified.

Barium meal study showed an ill-defined mass involving gastric fundus and extending into gastric cardia and lower gastroesophageal junction. A chest, abdominal, and pelvic contrast-enhanced computed tomography (CT) scans showed a 3.8 × 7.2 × 8.7 cm ill-defined mass, involving gastric fundus and extending into gastric cardia and lower gastroesophageal junction (Figures 1(a) and 1(b)). It was associated with multiple enlarged gastrohepatic lymph nodes; the largest measured 1.2 cm. There was no evidence of ascites or retroperitoneal or mesenteric lymphatic metastases. A CT-guided tissue biopsy was obtained and revealed DSRCT. Positron emission tomography (PET) (Figure 2(a)) and transverse-section PET-CT (Figure 2(b)) scans showed hypermetabolic fluorodeoxyglucose- (FDG-) avid mass lesion in gastric fundus, extending into gastric cardia and lower gastroesophageal junction. In addition, it was associated with a few hypermetabolic FDG-avid lesions in the gastrohepatic junction, indicating lymph node metastases. No evidence of distant metastasis was identified. In view of a probable neoplastic lesion, the Surgical Oncology team advised for surgical resection.

Patient underwent total gastrectomy with D2 lymphadenectomy, splenectomy, and antecolic Roux-en-Y esophagojejunal anastomosis. All resection margins were free from tumor cells. Metastatic tumor was found in five out of eighteen gastrohepatic lymph nodes. The common hepatic artery lymph nodes and the porta-hepatis lymph nodes were all reactive and negative for malignancy. The lymph nodes around the celiac access, splenic artery, and splenic hilum were involved and enlarged. Spleen showed no significant pathology. No evidence of ascites or peritoneal carcinomatosis was detected.

**Figure 3:**
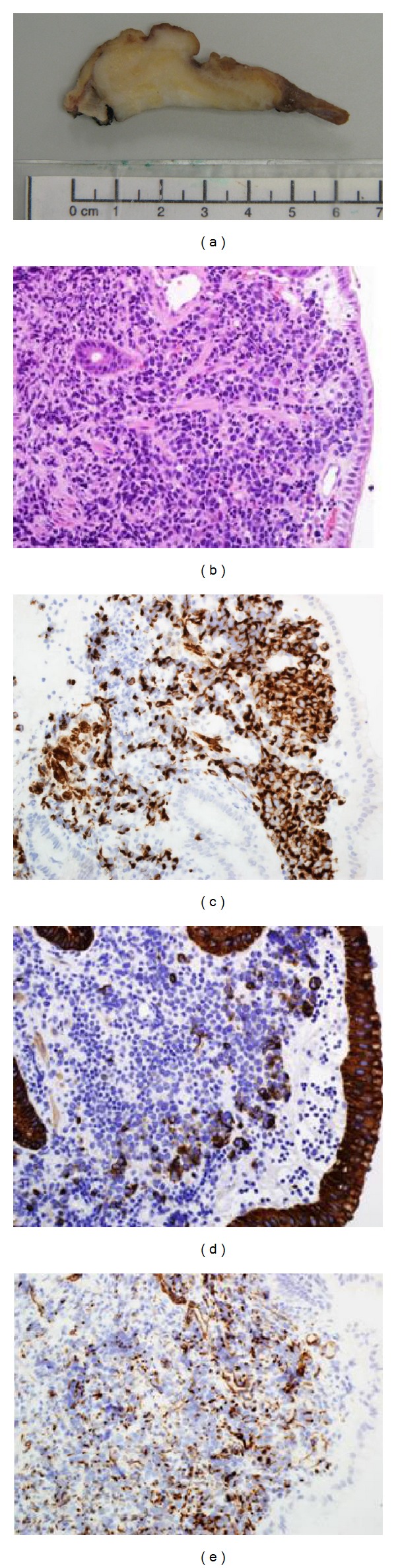
Desmoplastic small round cell tumor (DSRCT) of the resected gastric tumor mass. (a) Gross examination: cut section of gastric mass revealed a 2.5 × 4.5 × 6.5 cm, irregular, ulcerated, necrotic, and hemorrhagic mass involving the gastric fundus and extending into cardia and lower gastroesophageal junction. (b) Histopathological examination: showing intact epithelial mucosa and presence of sheets of small round blue cells separated by minimal desmoplastic fibrous stroma. (c) Desmin immunostaining: showing positive cytoplasmic staining in the tumor cells (magnification power: ×400). (d) AE1/AE3 (cytokeratin) immunostaining: showing focal mild positive staining in the tumor cells (magnification power: ×400). (e) WT1 immunostaining: showing dot-like positivity in the tumor cells (magnification power: ×400).

Macroscopically, the outer surface of the resected gastric mass was white, irregular, firm, and marked with several prominences. Cut section of gastric mass revealed a 2.5 × 4.5 × 6.5 cm, irregular, ulcerated, necrotic, and hemorrhagic mass involving the gastric fundus, and extending into cardia and lower gastroesophageal junction (Figure 3(a)).

Microscopically, histopathological examination of the resected gastric mass revealed intact epithelial mucosa and presence of sheets of small round blue cells separated by minimal desmoplastic stroma (Figure 3(b)). Immunohistochemical stains showed diffuse perinuclear staining pattern with desmin, but characteristic dot positivity was not prominent (Figure 3(c)). There was focal mild positive staining with AE1/AE3 (cytokeratin) (Figure 3(d)) as well as patchy dot positivity with WT1 (Figure 3(e)). There was negative staining with smooth muscle actin, calretinin, Myo-D1, myogenin, CD 45, chromogranin A, EMA, TTF1, synaptophysin, and S100. Strong positivity with desmin, AE1/AE3, and WT1 suggested that this lesion is most likely to be a case of DSRCT of stomach.

Furthermore, the resected specimen was analyzed by fluorescence in situ hybridization (FISH) technique and confirmed the diagnosis of DSRCT of stomach by identifying the characteristic EWS-WT1 gene fusion protein. Moreover, because of the unusual presentation, the resected specimen was sent to Mayo Clinic in Rochester, MN, USA, for diagnostic workup and results confirmed the abovementioned diagnosis (i.e., DSRCT of stomach).

Patient was discharged on the 10th postoperative day in a good condition and received no adjuvant radiotherapy or chemotherapy. A postoperative 3-month followup failed to show any recurrence.

## 3. Discussion

Desmoplastic small round cell tumor (DSRCT) is an extremely uncommon, highly aggressive, and malignant mesenchymal neoplasm of undetermined histogenesis [1]. It was first described by Gerald and Rosai in 1989 as a newly featured clinicopathologic entity [1]. It occurs most frequently in adolescent (young) males in their 2nd to 3rd decades of life, with a male-to-female ratio of approximately 4 : 1 [3]. Peritoneal cavity is the most frequent site of involvement and often associated with diffuse multiple peritoneal implants [4]. Extraperitoneal structures are occasionally involved and include lung [9], pleural serosa [10], parotid gland [11], ovary [12], paratesticular region [13], posterior cranial fossa [14], pancreas [15], soft tissue, and bone [16]. DSRCT has a particular increased propensity to diffusely spread along mesothelial-lined surfaces such as peritoneum and omentum (mesentery) [4, 5] with extensive lymphogenous and hematological metastases [2, 17]. Liver and lungs are the two most common regions involved in distant hematogenous metastases [2, 17].

Diagnosis of DSRCT is exceptionally challenging. Clinical signs and symptoms, as well as radiological findings, are nonspecific and similar to other intra-abdominal primary neoplasms [5, 8]. Such clinical signs and symptoms include nausea, vomiting, weight loss, abdominal pain, palpable abdominal mass, and ascites. Such radiological findings include calcifications, hydronephrosis, urinary obstruction, bowel obstruction, nodular peritoneal thickening, and retroperitoneal lymphadenopathy [5]. Despite the nonspecific radiological findings, chest computed tomography (CT) and whole-body positron emission tomography (PET) scans are regularly done for staging purposes [18].

Macroscopically, DSRCT appears as a large mass (sometimes reaching up to 20–40 cm in diameter) and is frequently accompanied by numerous peritoneal deposits. The outer surface is often smooth, firm, and bosselated (marked with bosses/eminences/protuberances). Cut section of DSRCT exhibits grey-tanned or whitish-grey surfaces associated with variable necrotic and hemorrhagic areas and occasionally myxoid changes [3–5, 19].

Microscopically, DSRCT is made up of distinct collections of small round blue tumor cells separated by plentiful desmoplastic fibrous stroma. The stroma may contain myxoid changes, cystic degenerations, or calcifications. Stroma can be mostly cellular or stromal although equal cellular and stromal proportions are also possible. Stroma typically contains spindle-shaped cells looking like fibroblasts or myofibroblasts. Rosette-like or palisading structures can be visualized. Furthermore, DSRCT is composed of small- to medium-sized tumor cells with ill-defined cell boarders, oval- to round-shaped hyperchromatic nuclei, indistinct nucleoli, and increased nuclear/cytoplasmic ratios. Variable degrees of mitotic and apoptotic figures are often seen. Eosinophilic cytoplasmic inclusion bodies can occasionally be identified [3–5, 19]. Cytological analysis is feasible from tissue biopsy and fine needle aspiration (FNA) specimens [20]. Ascitic and pleural fluid specimens are also of benefit especially if tissue biopsy or FNA specimens are difficult to obtain [20]. The rarity of DSRCT as well as its histopathological and cytological similarities to other small round blue cell tumors makes the diagnosis quite challenging.

Immunohistochemical techniques can be used to confirm accurate diagnosis of DSRCT. Immunohistochemically, DSRCT cells demonstrate positive staining to a wide variety of epithelial (cytokeratin and epithelial membrane antigen), mesenchymal (vimentin and desmin), and neural (CD56 and neuron-specific enolase) markers [3, 4]. This multiple antigen expression profile is largely characteristic for DSRCT and can be utilized to distinguish DSRCT from the other histologically related small round blue cell tumors [8].

DSRCT is associated with a distinctive chromosomal reciprocal translocation (t(11; 22)(p13; q12)), which fuses the Ewing's sarcoma gene on chromosome 22 to the Wilm's tumor type 1 gene on chromosome 11 [6]. This translocation results in EWS-WT1 gene fusion protein which is an altered transcriptional factor that modifies gene expression and eventually promotes uncontrolled proliferation of DSRCT cells. This translocation can be identified by reverse transcriptase-polymerase chain reaction (RT-PCR) method [21], fluorescence in situ hybridization (FISH) technique [22], or positive immunohistochemical staining to Wilm's tumor type 1 (WT1) protein [23]. The EWS-WT1 gene fusion protein serves as a disease-specific marker and yields a definitive diagnosis of DSRCT [6].

Currently, there are no standard (consensus) management protocols for DSRCT [2, 5]. This is attributed to its extreme rare incidence, high aggressive behavior, and lack of validated staging system for DSRCT [2]. In addition, all contemporary treatments have been only studied on a small series of patients or described in a limited number of case reports [5]. Aggressive surgical resection (debulking), radiotherapy, multiple-agent chemotherapy, and myeloablative chemotherapy with stem-cell rescue in selected circumstances have been shown to yield the best outcomes [7, 24, 25]. Newer novel treatment modalities which require further evaluations include cytoreductive surgery followed by continuous hyperthermic peritoneal perfusion (CHPP) with cisplatin [26], and Yttrium [90Y] microsphere treatment for liver metastasis in DSRCT [27]. Still, all contemporary treatment modalities for DSRCT are not curative and do not warrant long-term survival benefits [28].

In our case, the patient underwent only complete surgical resection (debulking) with no other modality, and, after a three-month followup, she presented with no recurrences. No decision was taken to use either chemotherapy or radiotherapy. In the opinion of our Medical Oncology team, chemotherapy remains controversial as the side effects of the treatment may overweigh the minimal gain in survival. In addition, the tumor is radioresistant and certainly the use of radiotherapy may cause toxicity and cause harm more than benefit.

DSRCT is an extremely uncommon, highly aggressive, and malignant mesenchymal neoplasm of undetermined histogenesis [1, 4]. Prognosis of DSRCT is extremely poor with an overall 5-year survival rate of 15% (median survival ranges from 17 to 25 months) [7]. This is because DSRCT is only slightly sensitive to radiotherapy and chemotherapy, and complete surgical excision is hardly ever achieved [29]. Nemours combined aggressive treatment regimens were attempted; however, neither curative outcome nor notable impact on long-term survival was achieved [28]. Further optimal management protocols have yet to be explored.
